# The effectiveness of shoe insoles for the prevention and treatment of low back pain: a systematic review and meta-analysis of randomised controlled trials

**DOI:** 10.1186/1471-2474-15-140

**Published:** 2014-04-29

**Authors:** Vivienne Chuter, Martin Spink, Angela Searle, Alan Ho

**Affiliations:** 1 Discipline of Podiatry, University of Newcastle, Sydney, NSW, Australia; 2 School of Psychology, Faculty of Science and Information Technology, University of Newcastle, PO Box 127 Ourimbah, Sydney, NSW 2258, Australia

**Keywords:** Systematic review, Meta-analysis, Low back pain, Insoles, Foot orthoses, Treatment, Prevention, Effectiveness, RCT

## Abstract

**Background:**

Low back pain (LBP) is a significant public health problem in Western industrialised countries and has been reported to affect up to 80% of adults at some stage in their lives. It is associated with high health care utilisation costs, disability, work loss and restriction of social activities. An intervention of foot orthoses or insoles has been suggested to reduce the risk of developing LBP and be an effective treatment strategy for people suffering from LBP. However, despite the common usage of orthoses and insoles, there is a lack of clear guidelines for their use in relation to LBP. The aim of this review is to investigate the effectiveness of foot orthoses and insoles in the prevention and treatment of non specific LBP.

**Methods:**

A systematic search of MEDLINE, CINAHL, EMBASE and The Cochrane Library was conducted in May 2013. Two authors independently reviewed and selected relevant randomised controlled trials. Quality was evaluated using the Cochrane Collaboration Risk of Bias Tool and the Downs and Black Checklist. Meta-analysis of study data were conducted where possible.

**Results:**

Eleven trials were included: five trials investigated the treatment of LBP (n = 293) and six trials examined the prevention of LBP (n = 2379) through the use of foot orthoses or insoles. Meta-analysis showed no significant effect in favour of the foot orthoses or insoles for either the treatment trials (standardised mean difference (SMD) -0.74, CI 95%: -1.5 to 0.03) or the prevention trials (relative risk (RR) 0.78, CI 95%: 0.50 to 1.23).

**Conclusions:**

There is insufficient evidence to support the use of insoles or foot orthoses as either a treatment for LBP or in the prevention of LBP. The small number, moderate methodological quality and the high heterogeneity of the available trials reduce the strength of current findings. Future research should concentrate on identification of LBP patients most suited to foot orthoses or insole treatment, as there is some evidence that trials structured along these lines have a greater effect on reducing LBP.

## Background

Low back pain (LBP) is estimated to affect up to 80% of adults and has significant associated socioeconomic and healthcare cost [1,2]. While the majority of acute episodes resolve within a six week time-frame [3], approximately 10% of cases progress to a chronic stage where symptoms remain present for three months or more [4]. Recurrence rates of LBP are high, with up to 44% of LBP sufferers experiencing a return of symptoms within a year, and 85% a recurrence over their life-time [5]. In up to 85% of LBP cases the mechanism of the pain is poorly understood and is classified as non-specific, i.e. of unknown origin [6]. The combination of unknown aetiology and high rates of recurrence make effective treatment difficult and the outcomes of specific interventions have been shown to be variable [7,8].

Foot function has been suggested to be an aetiological mechanism for the development of LBP [9,10]. Excessive foot pronation is proposed to produce prolonged internal rotation of the lower limb and disrupt sagittal plane forward progression of the body during gait [11]. This causes significant strain at the sacroiliac and lumbosacral joints contributing to the development of LBP [9-11]. A rigid high arched foot type has also been associated with the development of LBP [12]. This foot type diminishes the capacity for shock absorption by the foot and so pre-disposes to shock-induced pathology in the lower back [13]. In the presence of excessive or prolonged foot pronation, orthoses have traditionally been prescribed to reduce the extent and velocity of foot movement, correcting lower limb function and proximal posture [14,15]. In a rigid high arched foot type, shock-absorbing insoles are proposed to reduce the more proximal propagation of shock, subsequently reducing LBP [13].

Anecdotal evidence of significant short and long term pain reduction following intervention with customised foot orthoses [16,17] and prefabricated insoles [13] supports the role of functional foot devices in the treatment and prevention of LBP.

However these findings are not supported by previous systematic reviews [18] and foot orthoses and insoles are currently not considered in international and national clinical guidelines for the management of non-specific LBP [19]. Given the common use of insoles to treat LBP [20] and the lack of clear guidelines for use in clinical practice, further investigation is warranted. The aim of this analysis is to systematically review the current literature to determine for people with LBP or at risk of developing LBP, if insoles are effective in preventing or reducing LBP, compared to a sham or control treatment, and to evaluate study findings by meta-analysis where appropriate.

## Methods

### Search strategy

An electronic database search of title and abstract was conducted on the 16^th^ May 2013. The databases searched were MEDLINE (1950-May 2013), EMBASE (1980 – May 2013), the Cumulative Index to Nursing and Allied health Literature (CINAHL) (1982 – May 2013) and The Cochrane Library. Search terms used were back pain, backache, lumbago, shoe insert, shock absorber, insole, footwear, orthoses and orthotic (Additional file 1). No language, publication date or publication status restrictions were used. Hand searches of the reference lists of included trials, clinical guidelines and review articles were also performed.

### Eligibility criteria

Only published reports of randomised controlled trials or crossover trials that compared orthoses or insoles with no treatment or placebo treatment were included in this review. Included studies needed to investigate the prevention or treatment of non-specific LBP. For this review, the definition of LBP is pain and discomfort, localised below the costal margin and above the inferior gluteal folds, with or without leg pain. Non-specific LBP is further defined as LBP not attributed to recognisable, known specific pathology (e.g. infection, tumour, osteoporosis, ankylosing spondylitis, fracture, inflammatory process, radicular syndrome or cauda equina syndrome) [21]. Treatment trials were required to report an outcome measure for pain, while prevention trials had to report an incidence rate. Studies were excluded if the individuals involved had LBP caused by specific pathologies or conditions. Trials using insoles to treat limb length discrepancy (LLD) and pelvic obliquity were excluded as there is disagreement regarding whether LLD does predispose to musculoskeletal disorders and what magnitude of LLD is pathological [22]. Clinical trials that were not randomised or quasi-random were excluded.

### Study selection

One reviewer conducted the electronic searches (AS). Titles and abstracts were independently assessed by two reviewers (AS and VC). Disagreements were resolved by consensus and a third reviewer where necessary (MS). A standardised data extraction form was used to collect population characteristics, trial inclusion and exclusion criteria, intervention details, outcome data and overall conclusions from each trial.

### Quality assessment

Risk of bias in the individual studies was assessed using the Cochrane Collaboration Risk of Bias Tool [23]. Methodological quality was assessed using a modified version of the Quality Index as described by Downs and Black [24]. The final question (Question 27) dealing with statistical power was simplified to a score of 0 or 1, from the original score of 0 to 5. A trial received a score of 1 if a power or sample size calculation was stated, while a 0 was scored if no appropriate power calculation was described. Therefore, our modified index could result in a score between 0 and 28, with a higher score reflecting a superior methodological quality.

### Statistical analysis

All data analyses were performed using STATA version 12 software. A random effects model was used as the underlying assumptions are believed to be better suited to deal with the clinical heterogeneity of the back pain literature [25]. For trials assessing the prevention of LBP, the relative risk (RR) was computed for dichotomous data. With treatment of LBP trials, where different scales were used to measure continuous pain outcomes across trials, standardised mean differences (SMD) were calculated using approximations of the means/standard deviations [26] and Hedges g correction was used to reduce bias [27]. An effect size of greater than or equal to 0.8 was considered to represent a large clinical effect, 0.5 a moderate effect and 0.2 a small effect [28]. Statistical heterogeneity between studies was assessed by use of the I^2^ statistic, and for this review heterogeneity scores were interpreted as low (25%), moderate (50%), and high (75%) [29]. As heterogeneity tests tend to be lower in power, p < 0.1 is used to indicate heterogeneity rather than p = 0.05 [30]. It was predetermined bias would be assessed using a funnel plot, Egger’s test and the Copas Selection model.

## Results

### Study identification

**Figure 1:**
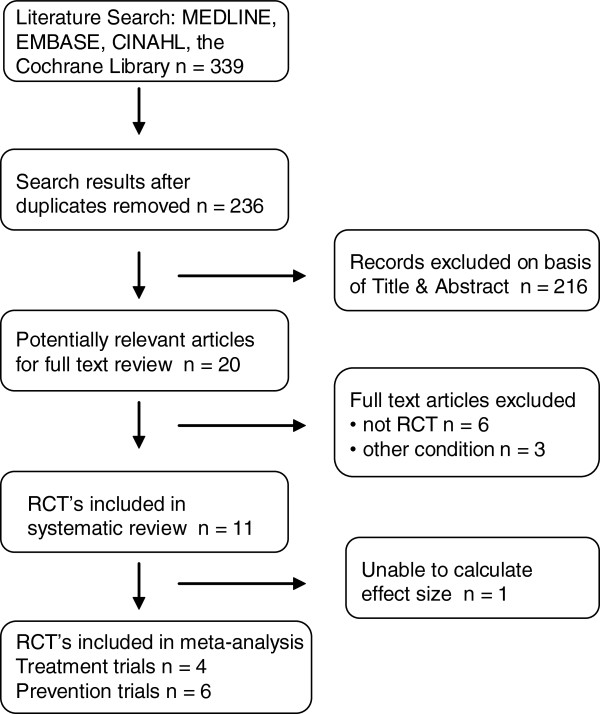
Flow diagram of systematic review inclusion or exclusion.

The initial database search resulted in a total of 339 citations of which 20 were appropriate for full review (Figure 1). After review, 11 trials were included (Tables 1 and 2) and nine were rejected on the basis of exclusion criteria (Additional file 2).

**Table 1:** Treatment studies: summary of included studies

**Author/s**	**Population**	**Participants**	**Insole & Comparison**	**Intervention**	**Time (weeks)**	**Quality score**
Almeida, 2009	Assembly line workers with work related musculoskeletal symptoms in the lumbar spine or lower limb	All female Age: 30.30 ± 7.09 Randomised: 27 Analysed: 27	Prefabricated (heat moulded Ethylene vinyl acetate) orthoses, individually customised **vs** Prefabricated simple insoles (placebo)	Participants were instructed to wear the insoles daily with the work uniform	8	78%
Basford, 1988	Office and laboratory workers whose job requires standing at least 75% of the day	All female Age: 39.00 ± 12.00 Randomised: 96 Analysed: 64	Prefabricated viscoelastic polyurethane orthoses, 1.3 mm at toe to 5 mm at heel (crossover trial)	Insoles were fitted to participants normal work shoes	5	57%
Cambron, 2011	Chronic LBP patients responding to advert	22 male, 28 female Age: 52.00 ± 16.00 Randomised: 50 Analysed: 46	Custom made polymer orthoses (flexible with arch support)	Participants given procedures for proper use of orthotics (but not detailed in article)	6 & 12	71%
Castro-Mendez, 2013	Chronic LBP patients with a Foot Posture Index (FPI-6) indicating at least one pronated foot	9 male, 51 female Age: 40.63 ± 14.63 Randomised: 60 Analysed: 51	Custom mould polypropylene & polyethylene orthoses in subtalar neutral position **vs** Flat polyester resin insole (placebo)	Participants were asked to wear the foot orthotics for at least 8 hours per day	4	79%
Shabat, 2005	Workers whose job required long distance walking & who suffered from LBP	25 male, 35 female Age: 39.14 Randomised: 60 Analysed: 57	Custom made viscoelastic polymer orthoses to support the foot **vs** Flat insole (placebo)	Participants were permitted to use insoles during work or non-work time	5	75%

**Table 2:** Prevention studies: summary of included studies

**Author/s**	**Population**	**Participants**	**Insole & Comparison**	**Intervention**	**Time (weeks)**	**Quality score**
Fauno, 1993	Soccer referees in 5 day competition	121 majority male Age: 35.90 ± 9.95 Randomised: 121 Analysed: 91	Prefabricated shock absorbing heel insoles, 8 mm thick vs No insole	Referees wore inserts in shoes for average of 870 minutes over 5 days	0.7	68%
Larsen, 2002	New military recruits starting training in a Danish regiment	145 male, 1 female Age: 18–24 Randomised: 146 Analysed: 121	Prefabricated (heat moulded) semi rigid orthoses **vs** No insole	Conscripts told to wear orthoses whenever wearing military boots	12	79%
Mattila, 2011	New military recruits starting service in Finland	All male Age: 19(18–29) Randomised: 220 Analysed: 220	Prefabricated polyethylene (heat moulded) ¾ length orthoses vs No insole	Participants told to use insoles in their ankle boots during daily service time	24	86%
Milgrom, 2005	New military recruits without a history of low back pain during basic training	All male Age: 18.80 ± 0.70 Randomised: 404 Analysed: 179	Custom semirigid biomechanical orthoses **vs** Custom soft biomechanical orthoses vs Simple shoe inserts (placebo)	Recruits monitored for compliance, but usage not stated in article	14	75%
Schwellnus, 1990	New military recruits doing standard training	All male Age: 18.50 ± 1.20 Randomised: 1511 Analysed: 1388	Prefrabricated flat neoprene insoles **vs** No insole	Recruits given instructions to wear insoles daily in the standard footwear	9	68%
Tooms, 1987	Senior nursing students whose work required prolonged standing or walking	Sex unknown Age: 22.85 ± 5.35 Randomised: 100 Analysed: 100	Prefrabricated viscoelastic insoles **vs** No insole	Participants requested to wear insoles in their regular work shoes	5	64%

### Characteristics of included studies

Five of the studies, involving 293 participants with an age range of 30 to 51 years, investigated the use of insoles for the treatment of LBP (Table 1) [31-35]. Two of these trials [31,32] used only female participants (n = 123). The remaining six studies, involving 2379 participants with a relatively younger mean age range of 18 to 36 years, examined insoles in the prevention of LBP (Table 2) [35-41]. The majority of these trial participants were male military recruits (n = 2281). The materials and design features of the foot orthoses and insoles used as interventions varied widely between the studies (Table 1). The time period for the use of orthoses was also variable, ranging from 5 days to 24 weeks. Four of the studies issued the comparison group with sham insoles [31,34,35,39], another two trials were crossover or wait list designs [32,33], while the remaining five trials provided no intervention to the control group [36-38,40,41]. Three of the treatment trials used a visual analog scale to measure pain [33-35], and the other two used an ordinal scale [31,32]. Three of the prevention trials measured LBP incidence via reported LBP and at least a day off duty [37,38,40], two trials used a self-report questionnaire plus a medical assessment [36,39], while the final trial used a self-report questionnaire only [41].

### Study quality and bias

The modified Downs and Black [24] quality index scores ranged from 57 to 86% (mean = 73%) (Additional file 3). The majority of studies scored highly on reporting of the interventions used and outcome measures. Only three trials [32,33,39] provided details of adverse events related to insole or orthotic use. The risk of bias as assessed with the Cochrane Collaboration risk of bias tool (Additional file 4) was generally low or unclear. The greatest potential source of bias was associated with blinding.

### Synthesis of results

As the characteristics of the treatment trials (n = 4) and the prevention trials (n = 6) were similar, meta-analysis of the two separate groups was considered appropriate. One treatment study [32] was excluded from the meta-analysis because we were unable to contact the authors for data that would allow calculation of effect sizes. Statistical analysis to assess the risk of publication bias was not used due to having fewer than 10 trials in the meta-analysis meaning test power is usually too low to distinguish chance from real asymmetry [42].

### Insoles or foot orthoses versus No intervention or placebo insoles for the treatment of LBP

**Figure 2:**
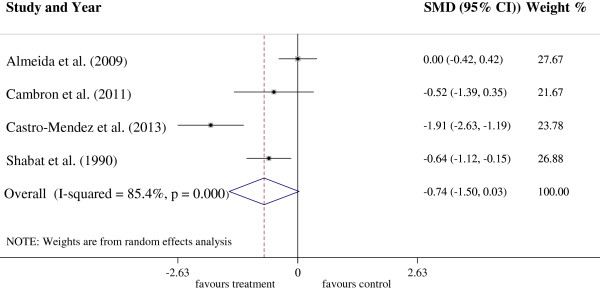
Forest plot of the treatment of LBP with insoles or foot orthoses intervention.

Four trials with 197 participants were included in this treatment subgroup meta-analysis (Figure 2). The analysis demonstrated a non-significant reduction in LBP between groups (SMD = 0.74, CI 95%: -1.5 to 0.03) in favour of foot orthoses, although a high amount of heterogeneity (I^2^ = 85.4%, p < 0.01) was present. The treatment group generally showed a positive trend, with three of the four trials reporting results that favour the insole intervention over the control treatment [33-35]. However only two of these trials reported results with statistical significance (Castro-Mendez et al. [34] (ES = -1.91, CI 95%: -2.63 to -1.19, p < 0.01)) and Shabat et al. [35] (ES = -0.64, CI 95%: 1.12 to -0.15, p = 0.01)).

### Insoles or foot orthoses versus No intervention or placebo insoles for the prevention of LBP

**Figure 3:**
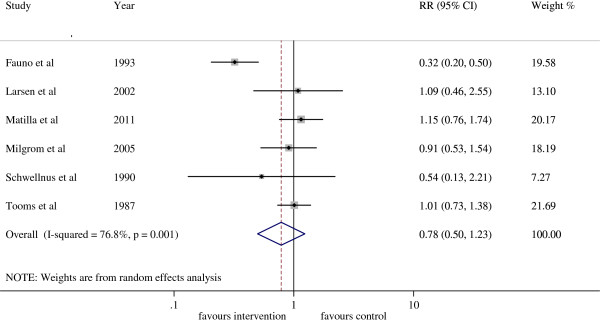
Forest plot of prevention of LBP with insoles or foot orthoses intervention.

Six trials with 2379 participants were included in this prevention subgroup meta-analysis (Figure 3). The analysis demonstrated a 22% reduction in the risk of developing LBP with the use of foot orthoses or insoles (RR = 0.78, CI 95%: 0.50 to 1.23) compared to the control group, however these results were not statistically significant and a high level of heterogeneity was present (I^2^ = 76.8%, p < 0.01). Of the six prevention trials, only one low quality trial reported results with statistical significance [36]. The results of one trial [39] may have been affected by the high drop out rate, with only 45.5% and 67.5% of the participants in the two intervention groups completing the trial using their orthoses.

## Discussion

Our review identified five trials that assessed orthoses for the treatment of LBP, and another six trials using orthoses for the prevention of LBP. No statistically significant effect for the use of insoles or foot orthoses is seen for either prevention trials or treatment trials.

Meta-analysis of the efficacy of foot orthoses and insoles for the treatment of LBP did not demonstrate any significant effect of treatment. The results are trending in a positive direction but only two of the trials reach statistical significance [34,35]. Of note, the study reporting the largest effect size targeted participants with a pronated foot posture [34]. This may be due to the participants either having a more homogenous response to orthoses or having a similar functional aetiology for their LBP resulting in a greater treatment effect. Currently there is insufficient evidence to recommend the use of insoles or foot orthoses in the treatment of LBP and larger RCTs over longer periods are required.

The results for the use of insoles or foot orthoses in the prevention of LBP are less positive. Only one low quality trial reported foot orthoses or insoles to be effective in preventing LBP [36]. However, most of the prevention trial participants were male military recruits with a mean age < 20 years with no pre-existing conditions that would preclude them from military duty. This limits the generalizability of these findings to the wider population. Furthermore this group is not representative of people typically at high risk for LBP, with international data indicating that the incidence of LBP is highest in the third decade and that prevalence increases with age, peaking in the 6^th^ decade of life [43,44]. Consequently the lack of external validity of the prevention trials brings in to question current evidence that insoles or foot orthoses are not effective in the prevention of LBP.

This review identified limited evidence for the effectiveness of foot orthoses or insoles in the treatment and prevention of LBP. However, it has been demonstrated that foot orthoses and insoles have been effective as a mechanical therapy in other musculoskeletal conditions including patellofemoral pain syndrome [45], medial compartment knee osteoarthritis [46] and femoral stress fractures [47]. A contributing factor for this difference may be the methodology of the included studies. The rationale behind most studies was that orthoses may mitigate the effects of high or low arched feet on the lower limb kinetic chain and so prevent or reduce LBP. However, only one trial [34] assessed foot type and included only participants with pronated (low arched) feet. Consequently in most trials it is unknown if the intervention was addressing the causative mechanism or not. Another contributing factor may be the large degree of heterogeneity between the studies with considerable variation in the trial populations, periods of use of the orthoses, and materials and design features of the orthoses.

### Research recommendations

LBP remains a considerable health problem in all developed countries [19,48] and the failure of the current evidence to conclusively identify effective interventions to improve clinical outcomes and reduce associated healthcare costs has led to calls for more targeted trials [49,50]. Proponents argue that better outcomes may be achieved by classifying patients into subgroups and prescribing treatment relevant to their clinical presentation. Clinical prediction rules (CPRs) are defined as decision making tools for clinicians that utilise information gained from the history and examination, and can be used to guide a therapeutic course of action [51]. Studies using CPRs to guide the choice of physical therapy or exercise plans for LBP treatment have reported better functional outcomes if the participants receive a treatment matched to their subgroup compared to an unmatched treatment [52,53].

Our analysis of the treatment trials also supports this proposition. Only the Castro-Mendez [34] trial used an abbreviated CPR to guide treatment options and they reported the largest effect size in the reduction of LBP and disability using customised foot orthoses. An inclusion criteria for this trial was foot pronation which has been proposed as a contributing factor to LBP [9,10]. Foot orthoses has been reported as an effective treatment strategy for pronation [14]. In addition, the majority of these participants were female (female = 43, male = 8) and research has shown a higher prevalence of LBP in females with pronated feet [54]. However, the researchers used pronation as the only factor from the clinical examination in their CPR and further research would be required to develop a robust CPR for the prescription of insoles for patients with LBP. As the other trials reviewed did not attempt to provide a matched treatment to their participants, it is possible that the effect size of the insoles or foot orthoses has been underestimated.

### Limitations

Although this review was designed to be comprehensive with a robust search strategy, it is possible that that not all studies were identified. In addition, only RCTs were considered to have appropriate levels of evidence, so studies with lesser levels of evidence such as case series have been excluded. The strength of evidence in this review is impacted by the small number of trials identified and the low to moderate methodological quality of included trials. Only two of the higher quality studies [34,38] reported power calculations to enable detection of a clinically important difference between the interventions. Further, the control in four of the trials [31,34,35,39] was a sham insole which would not have provided any functional support to the foot but may have induced a placebo effect. Finally, our analysis only investigated the effect that insoles or foot orthoses had on pain. We did not assess other measures, such as quality of life and psychological health, which are commonly affected in people with LBP, and which may be improved by insoles or foot orthoses.

## Conclusions

There is insufficient evidence to support the use of foot orthoses or insoles as either a treatment for LBP or in the prevention of LBP. The small number, moderate methodological quality and the high heterogeneity of the available trials reduce the strength of current findings. At risk populations should be targeted in future trials examining LBP prevention. Future research for LBP treatment should concentrate on variables from the patient history, physical examination or simple diagnostic tests that may assist in classification of LBP patients most suited to a foot orthoses or insole intervention, as there is some evidence that trials structured along these lines have a greater effect on reducing LBP.

## Competing interests

The authors declare that they have no competing interests.

## Authors’ contributions

VC contributed to the conception and design of the review; analysis and interpretation of data; and drafting and revising of the manuscript. MS contributed to the design of the review; analysis and interpretation of data; and drafting and revising of the manuscript. AS contributed to the acquisition, analysis and interpretation of data; and drafting and revising of the manuscript. AH contributed to the analysis of data; application of statistical techniques; and drafting and revising of the manuscript. All authors read and approved the final manuscript.

## Pre-publication history

The pre-publication history for this paper can be accessed here:

http://www.biomedcentral.com/1471-2474/15/140/prepub

## Supplementary Material

Additional file 1 **Database Search strategy.** File shows a detailed description of the database search for MEDLINE, CINAHL, EMBASE and The Cochrane Library. Click here for file

Additional file 2 **Excluded studies.** File is a table showing the exclusion grounds for articles excluded after full-text assessment. Click here for file

Additional file 3 **Quality Index Scores.** File is a table showing the individual Downs and Black quality index scores for each included article. Click here for file

Additional file 4 Cochrane risk of bias table. Click here for file
