# Supplementary material for: The effectiveness of shoe insoles for the prevention and treatment of low back pain: a systematic review and meta-analysis of randomised controlled trials
Source: BMC Musculoskelet Disord. 2014 Apr 29;15:140. doi: 10.1186/1471-2474-15-140 (PMC4107719; doi:10.1186/1471-2474-15-140)
Supplement: Additional file 1 — Database Search strategy. File shows a detailed description of the database search for MEDLINE, CINAHL, EMBASE and The Cochrane Library. [file 1471-2474-15-140-S1.doc]

**Additional File 1**: Description of search strategy. A detailed description of the database search

| **Database** | **Time Span** | **Search Strategy** |
| --- | --- | --- |
| Cochrane Central Register of Controlled Trials | Issue 4 of 12, April 2013 | ((back and pain) or backache or lumbago) AND (shoe or insert or (shock and absorber) or insole* or footwear or orthoses or orthotic |
| MEDLINE | 1950 - May 2013 | 1. ((back and pain) or backache or lumbago)  2. (shoe or insert or (shock and absorber) or insole$ or footwear or orthoses or orthotic$)  3. 1 and 2  4. limit 3 to (humans and randomized controlled trial) |
| EMBASE | 1974 – May 2013 | 1. ((back and pain) or backache or lumbago)  2. (shoe or insert or (shock and absorber) or insole$ or footwear or orthoses or orthotic$)  3. 1 and 2  4. limit 3 to (human and (clinical trial or randomized controlled trial or controlled clinical trial)) |
| CINAHL | 1982 – May 2013 | 1. (back and pain) or backache or lumbago  2. shoe or insert or (shock and absorber) or insole$ or footwear or orthoses or orthotic$  3. 1 and 2  4. Narrow by SubjectMajor: Foot Orthoses, Back Pain, Low Back Pain |
