# Supplementary material for: The effectiveness of shoe insoles for the prevention and treatment of low back pain: a systematic review and meta-analysis of randomised controlled trials
Source: BMC Musculoskelet Disord. 2014 Apr 29;15:140. doi: 10.1186/1471-2474-15-140 (PMC4107719; doi:10.1186/1471-2474-15-140)
Supplement: Additional file 2 — Excluded studies. File is a table showing the exclusion grounds for articles excluded after full-text assessment. [file 1471-2474-15-140-S2.doc]

**Additional File 2**: Excluded studies. A table showing the exclusion grounds for articles excluded after full-text assessment.

|  | **Author** | **Title** | **Grounds for Exclusion** |
| --- | --- | --- | --- |
| 1 | Baxter | Lower Limb Injuries in Soldiers: Feasibility of Reduction Through Implementation of a Novel Orthotic Screening Protocol. Military Medicine 2011;176(3):291-6 | Orthoses issued according to protocol (not random) |
| 2 | Dananberg | Chronic low-back pain and its response to custom-made foot orthoses. Journal of the American Podiatric Medical Association 1999;89:109-17 | Non randomised trial |
| 3 | Defrin | Conservative correction of leg-length discrepancies of 10mm or less for the relief of chronic low back pain. Archives of Physical Medicine and Rehabilitation 2005;86:2075-9 | Limb Length Discrepancy correction |
| 4 | Fann | The impact of structural therapy on functioning and pain in chronic pain patients: A pilot study. Journal of Back and Musculoskeletal Rehabilitation 2007; 20:1-9. | Pelvic Obliquity correction |
| 5 | Ferrari | Responsiveness of the Short-Form 36 and Oswestry Disability Questionnaire in chronic nonspecific low back and lower limb pain treated with customized foot orthotics. Journal of Manipulative & Physiological Therapeutics 2007;30:456-8 | Non randomised trial |
| 6 | Golightly | Changes in pain and disability secondary to shoe lift intervention in subjects with limb length inequality and chronic low back pain: a preliminary report. Journal of Orthopaedic & Sports Physical Therapy 2007;37:380-8 | Limb Length Discrepancy correction |
| 7 | Landsman | Scientific assessment of over-the-counter foot orthoses to determine their effects on pain, balance, and foot deformities. Journal of the American Podiatric Medical Association 2009;99:206-15 | Non randomised trial |
| 8 | Sobel | The Effect of Customized Insoles on the Reduction of Postwork Discomfort. Journal of the American Podiatric Medical Association 2001;91:515-20 | Non randomised trial |
| 9 | Wosk | Low back pain: conservative treatment with artificial shock absorbers. Archives of Physical Medicine & Rehabilitation 1985;66:145-8 | Non randomised trial |
