# Supplementary material for: The effectiveness of shoe insoles for the prevention and treatment of low back pain: a systematic review and meta-analysis of randomised controlled trials
Source: BMC Musculoskelet Disord. 2014 Apr 29;15:140. doi: 10.1186/1471-2474-15-140 (PMC4107719; doi:10.1186/1471-2474-15-140)
Supplement: Additional file 4 — Cochrane risk of bias table. [file 1471-2474-15-140-S4.docx]

Additional File 4: Risk of bias assessment table

|  | **Sequence Generation** | **Allocation Concealment** | **Blinding of participants and personnel** | **Blinding of outcome assessment** | **Incomplete outcome data** | **Selective reporting** |
| --- | --- | --- | --- | --- | --- | --- |
| **Almeida 2009** | 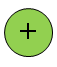 | 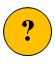 | 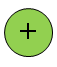 | 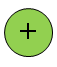 | 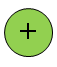 | 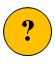 |
| **Basford 1988** | 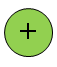 | 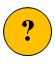 | 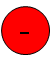 | 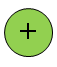 | 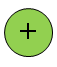 | 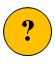 |
| **Cambron 2011** | 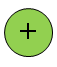 | 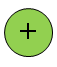 | 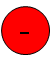 | 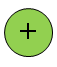 | 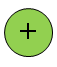 | 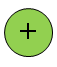 |
| **Castro-Mendez 2013** | 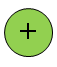 | 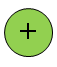 | 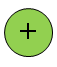 | 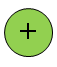 | 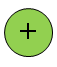 | 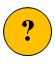 |
| **Fauno 1993** | 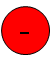 | 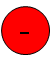 | 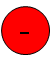 | 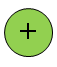 | 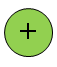 | 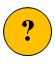 |
| **Larsen 2002** | 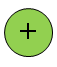 | 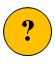 | 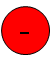 | 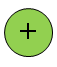 | 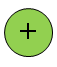 | 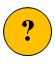 |
| **Mattila 2011** | 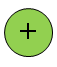 | 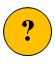 | 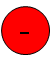 | 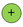 | 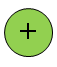 | 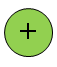 |
| **Milgrom 2005** | 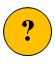 | 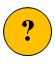 | 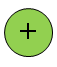 | 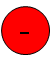 | 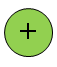 | 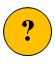 |
| **Schwellnus 1990** | 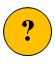 | 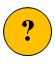 | 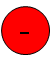 | 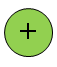 | 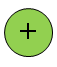 | 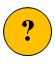 |
| **Shabat 2005** | 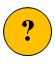 | 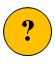 | 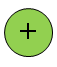 | 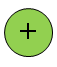 | 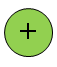 | 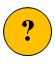 |
| **Tooms** | 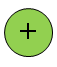 | 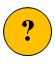 | 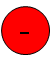 | 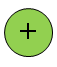 | 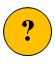 | 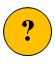 |


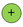
 low risk of bias,
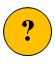
 unclear risk of bias,
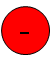
 high risk of bias as established applying the Cochrane Collaboration tool for assessing risk of bias
